# Bibliographical Notices

**Published:** 1846-09

**Authors:** 


### BIBLIOGRAPHICAL NOTICES.

*An Inquiry into the Physiological and Medicinal properties of the Aconitum Napellus; to which are added Observations on several other Species of Aconitum.* By Alexander Fleming, M. D., President of the Royal Medical Society of Edinburgh. 8vo. pp. 160. John Churchill: London, 1845.

This essay was presented as an "Inaugural Dissertation," and "obtained from the Seratus Academicus of Edinburgh a Gold Medal at the Graduation in 1844," and was afterwards printed in accordance with the recommendation of the Medical Faculty, which is alone high testimony to its merits.

It is seldom, indeed, that we have the opportunity of noticing a work so original in its character and yet so practical in its tendency; coming, too, from a youthful member of the profession—one just entering its threshold.

Dr. Fleming, in his inquiry into the physiological and medicinal properties of the Aconitum Napellus, appears to have followed closely in the footsteps of Magendie, Orfila, and other judicious investigators, by the institution of experiments under his own observation, repeated and varied until the observations were sufficiently numerous to authorize him to assume the results as proper bases for therapeutical guidance. Although it be true that the physiological actions excited by a medicine during a condition of health, are often very different from its effects in disease, still, the careful observation of such effects affords the most probable guide to its administration in the absence of more positive knowledge; and when the effects in disease are found to correspond with those manifested by experiments tried during health, we seem to have arrived at the best evidence of which the case is susceptible.

Contrary to what is commonly observed, Dr. Fleming says "the properties of the Aconitum Napellus do not suffer change by *cultivation*, and, in all probability, are little influenced by climate."

Dr. F.'s experiments with the various parts of the plant showed that the tuber is the most powerful part, and the flowers next in activity. "The tuber is more active immediately after the period of flowering, than at any other time. It has then attained its largest size. Soon afterwards, the bud of the plant of the succeeding year begins to shoot out from its apex, during the gradual development of which, a perceptible diminution in its activity may be detected. The tuber of this year becomes the root stock of the next, in the autumn of which it rots and dies. Thus the root is biennial, and the stem annual.

The activity of the leaves continue from their first appearance till the seeds begin to form, after which it quickly diminishes, though the leaves are at that period even more matured in size than before."

The author concludes, therefore: "*First*, That the root is the most eligible part of the plant for medicinal use, both on account of its greater activity, and also from the ease with which it may be obtained in large quantities; and,

*Secondly*, That the leaves ought to be gathered before or during the flowering season, and the tuber soon after it."

*Physiological Action of the A. Napellus on Animals.*—Aconite, when introduced into the system of one of the lower animals, produces, in the first instance, weakness of the limbs and staggering. The breathing then becomes either slightly accelerated, or slow and labouring. The paralysis increasing, the animal is at last unable longer to support itself, and lies down upon its side, with the extremities stretched out in a relaxed state. The general sensibility of the surface is impaired, and, towards the fatal termination, is altogether lost. Blindness, to a greater or less extent, soon supervenes; the breathing becomes gradually slower and more imperfect; and after a few spasmodic twitches, death by *Asphyxia* ensues.

On examination of the body immediately after death, the heart is found beating with considerable strength, nor does its action cease for some time. The peristaltic motion of the intestines also continues. The irritability of the voluntary muscles is impaired, as is evinced by their being less easily excited to contraction by mechanical irritation, than is usually the case, although they still respond readily to galvanism. General venous congestion exists; the right side of the heart is distended; there is engorgement of the venæ cavæ, of their tributary veins, and frequently of the brain; venous blood may usually be detected in the left side of the heart and in the aorta. The blood coagulates, and the muscles become rigid as usual.

"One of the most characteristic symptoms of the action of the poison," according to our author, "is *muscular paralysis.* This affects, first, the muscles of the extremities, afterwards those of the trunk, and finally proves fatal, by extension to the muscles of respiration."

Although the author concludes from his experiments that the A. Napellus exerts a *sedative* action on the heart, by which the pulse becomes weaker and less frequent; yet, "when the dose is large, and proves quickly fatal, the heart is comparatively little affected; and, certainly, death is not produced by failure of the circulation."

Dr. F. has "never observed Aconite produce such a change in the vascularity of any part to which it was applied, as was sufficient to prove an *irritant action.*" "The rapidity and intensity of the remote effects of Aconite, are in direct proportion to the absorbing powers of the part to which it is applied, producing no effect when placed in contact with the skin, and acting with less energy when taken into the stomach than when introduced into the serous cavity, or into the cellular tissue. It acts with the greatest energy and rapidity, when introduced directly into the circulation."

From several experiments performed on the *Infusoria*, Dr. F. ascertained that Aconite is poisonous to them in common with other animals.

"*Lastly*"—he observes—"Numerous experiments justify me in affirming, that Aconite is a direct sedative poison to *vegetables*. A healthy plant, whose root is introduced into water impregnated with it, speedily begins to fade; its leaflets lose all their original freshness, hang over the edge of the glass, and soon shrivel and die."

The most interesting remarks of the author relate to the physiological action of the Aconitum Napellus on *man*. We shall offer no apology for giving his conclusions on this part of his subject in his own words.

*Physiological Action of the Aconitum Napellus on Man.*

"Division I.—Topical Action. Aconite, when applied externally, acts as a *direct sedative to the nerves of sensation*. When a small piece of the root is chewed, the flow of saliva is increased, and heat and tingling, followed by numbness, are felt in the lips and tongue. A sense of swelling or distension is also perceived in these parts, and the motion of the lips is less free than natural. The effects of the alkaloid are similar, but more intense.

If a larger piece of the root be chewed, a small portion of which is swallowed, the palate and throat are affected, and the uvula feels as if elongated and in contact with the tongue. A sense of constriction of the throat even to a painful degree, is also occasionally experienced. When any preparation of the drug is applied to the pituitary and conjunctival membranes, similar effects are produced as on the lips, while their respective secretions are much increased in quantity. When the pituitary membrane is affected, frequent sneezing is induced, and a sensation resembling that which attends a severe cold in the head is perceived. These effects continue for a longer or shorter period, according to their intensity, sometimes for many hours.

The alkaloid, or the tincture of the root, when applied to the skin, causes intense heat and tingling, to which succeed numbness and a sense of tightness or dragging. In consequence of the mechanical barrier presented to the action of the drug by the cuticle, the skin requires to be briskly rubbed with the preparation for a considerable length of time. Slight redness is thus produced, but no more than may be accounted for by the simple act of friction.

Aconite impairs the *common sensibility* of the part to which it is applied. Thus, when the lips are severely affected, their mutual pressure is not perceived and very little pain is experienced on their being pinched. That it also impairs the function of the nerves of *special sensation* is shown by the fact, that when the tongue or nostrils are affected, their respective senses are much blunted. Its sympathetic action on the *retina* is remarkable. When applied to one of the temples or one side of the forehead, more or less blindness of the same side is frequently produced.

The topical effects of Aconite are, as might readily be supposed, most marked when it is applied to a surface abundantly supplied with nerves. Thus if a piece of the root be slightly chewed and then rubbed vigorously on the hard palate, numbness and tingling will come on with their usual intensity in the lips and tongue, while they will hardly be perceptible in the palate. In the same way, the tips of the fingers are more readily affected than any other part of the integuments.

Its local action on the *muscular system* is also of a *direct sedative* character. * * * *

Its topical application is, as far as I have seen, *unaccompanied either by pain, redness, or swelling*, even when the physiological effects are developed to the fullest extent. Some have believed it capable of exerting a stimulant action on the vascular system, an opinion which, as has already been shown, meets with no support from my experiments on animals. It has been asserted that the peculiar sensations produced by chewing the root are accompanied by slight inflammatory action; but careful observation has convinced me that this is not the case. Nor is the increase in the lachrymal, pituitary, and salivary secretions, already noticed, to be regarded as the effect of any irritant action of the drug, but simply as the fruit of the sympathy existing between the nerves of the mucous membranes and the secreting organs. Other authors assert that the leaves, when applied to the skin, produce an erysipelatous inflammation, with an eruption of vesicles. This experiment I have repeatedly tried without once meeting with such a result. * *

When the conjunctiva is slightly painted with the ointment of Aconitina, *contraction of the pupil* speedily takes place, and continues for several hours. Mr. Pereira has observed contraction of the pupil in some amaurotic cases of several year's standing, and where the iris underwent no change on exposure to strong light. When, on the other hand, the ointment of the alkaloid, or the tincture of the root, is applied to the temple or forehead, the pupil occasionally becomes *dilated.* I have only seen this latter occurrence in two cases, in both of which it was accompanied by partial blindness of the same eye. These phenomena are either effected by reflex action through the fifth and third nerves, or perhaps by imbibition ; but why such entirely opposite effects should ensue in the two cases, it is difficult to understand.

I may mention that I have several times anointed the eyelids with the alcoholic extract, in the same way as Belladonna is usually applied for the purpose of producing dilatation of the pupil, but without the slightest effect ; a circumstance perhaps accounted for by the fact that the action of Aconite on the skin is only developed after considerable friction has been employed.

Division II. — Physiological Action on Man in small and medicinal doses.—I shall in the first place, take a general view of the ordinary effects of Aconite in small or medicinal doses; and, for the sake of convenience, shall consider these under four degrees of operation.

*First Degree of Operation.*—In the course of twenty minutes or half an hour, after the exhibition of five minims of the tincture, a feeling of warmth in the stomach is usually experienced, which is occasionally accompanied by slight nausea and oppression of the breathing. After the lapse of thirty or forty minutes, this sense of warmth is diffused throughout the body, and, in a few minutes more, is attended by numbness, tingling, and a sense of distension of the lips and tongue. There is also tingling at the tips of the fingers, and a peculiar sensation is felt at the roots of the teeth. The feeling of warmth soon disappears, but the numbness and tingling of the lips and fingers continue for a period varying from one to three hours. Slight muscular weakness is generally experienced, with indisposition for exertion either mental or corporeal. In about half an hour more, the pulse is found to be diminished in strength ; and in another hour, both the pulse and the respiration have become less frequent. Thus, a pulse which, in the normal state, beats seventy-two in the minute, will, by that time, have fallen to about sixty-four, and the respirations, supposing them to have been eighteen, to fifteen or sixteen.

*Second Degree of Operation.*—Should a dose of ten minims be given at first, or the first dose of five minims be succeeded in two hours by another of equal amount, these symptoms supervene more rapidly, and with greater severity. The tingling extends along the arms, and the sensibility of the surface is more or less impaired. In an hour and a half, the pulse will probably have fallen to about fifty-six beats in the minute, and become smaller and weaker than before, still maintaining, however, perfect regularity. The respirations will have diminished to about thirteen, presenting, at the same time, a slow labouring character. Great muscular debility is now experienced; and giddiness, with confusion of sight, comes on when the erect posture is assumed. The individual sinks into a lethargic condition, evinces great disinclination to be disturbed, although he rarely falls asleep, and complains much of chilliness, particularly in the extremities, which are cold to the touch. These phenomena continue in their full intensity from three to five hours, when they gradually disappear, a sensation of langour, which lasts for several hours more, alone remaining.

This is the utmost extent to which I would recommend the physiological effects of Aconite to be carried, in order to obtain, with safety and success, its therapeutic action.

*Third Degree of Operation.*—On the administration of five minims more, two hours subsequent to the last dose, the sense of warmth, and the numbness and tingling, again spread rapidly over the body. The sensibility of the surface is still farther diminished; lancinating pains in the joints are occasionally complained of; the headache, vertigo, and dimness of vision, are aggravated; the countenance grows pale and anxious; the muscular feebleness increases; the voice becomes weak, and the individual is frequently impressed with the dread of approaching dissolution. Occasionally, the pulse is reduced still further in strength and frequency, perhaps falling to 40, or even 36 beats per minute, but still maintaining its regularity. More frequently, however, it rises to 70 or 80, and becomes small, weak, and probably more or less irregular. The respiratory movements are also irregular, being either short and hurried, or deep and sighing. The surface is moist, and still farther reduced in temperature. Sickness may now come on; and, if formerly present, is much aggravated, and probably attended by vomiting. These symptoms do not entirely subside for one or two days.

*Fourth Degree of Operation.*—If the administration be carried further, the symptoms assume a more alarming character. The countenance becomes pale and sunken; froth issues from the mouth, and the prostration increases. Two patients thus affected stated, that they felt as if dying from excessive loss of blood. Consciousness usually remains; or there may be slight wandering delirium, as occurs also after profuse hæmorrhage. The voice is whispering, or is altogether lost. The pulse becomes still smaller, weaker, and more irregular; and the breathing more imperfect. The surface is colder than before, and is covered with a clammy sweat.

When the action of the drug is carried to a fatal extent, the individual becomes entirely blind, deaf, and speechless. He either retains his consciousness to the last, or is affected with slight wandering delirium; the pupils are dilated; general muscular tremors, or even slight convulsions, supervene; the pulse becomes imperceptible, both at the wrist and heart; the temperature of the surface sinks still lower than before; and at length after a few hurried gasps, death by *syncope* takes place.

It must be borne in mind, that these symptoms do not, on all occasions, occur in the uniform manner in which they have now been described. On the other hand, some of them may be entirely absent; while others not yet mentioned, but to which I shall afterwards allude, may appear. In some cases, also, great insensibility to the influence of the remedy is manifested.

In administering it, it is proper to begin with a small dose, which must be increased or repeated, until the physiological effects of the drug, of the intensity described under the second degree of operation, have been produced."

After these general observations, the author proceeds to detail the effects of the article on the different systems of organs.

I. On the Cerebro-Spinal System.—Aconite, he remarks, in all doses, exerts a remote action on the nervous system, and this it does in three ways.

1. *Primarily—by its direct or specific action when conveyed to it by the blood.*—This is purely *sedative* in its nature, from the first, and the closest scrutiny fails to detect any symptom calculated to warrant the belief that a primary stimulant action is exerted.

2. *Secondly—by its sedative action on the circulation.*—When this is fully developed, the flow of arterial blood to the brain is much diminished; an occurrence which impairs the energies of that organ, in the same way as *excessive loss of blood;* between which, and the action of Aconite, there exists a strong analogy.

3. *Aconite acts on the Brain and Spinal Cord:—Secondarily,* by producing *engorgement of their venous system.* This mode of action is never developed but when poisonous doses are given. The engorgement of the nervous centres is a part of the general venous congestion, consequent on the difficulty of breathing.

II. "On the Muscular System. The action of Aconite on the muscular system is *directly and powerfully sedative.* In the second degree of operation, the patient complains of general weakness, which, in the third, may advance to a feeling of complete prostration. When this is excessive, it may be accompanied by partial or complete loss of voice. Muscular tremors and nervous twitches of the limbs are sometimes, though rarely, observed. The muscular debility endures for a period, varying, according to its intensity, from a few hours to several days."

III. "ON THE VASCULAR SYSTEM. Aconite exerts a *direct sedative* influence on the vascular system, reducing, more or less, according to the dose administered, the strength, volume, and, in the first instance, the frequency of the pulse. The diminution in frequency varies much, *cæteris paribus*, with the individual; the pulse, in some cases, not falling below 60, and, in others, sinking so low as 48, 40, and even 36. As a general rule, it maintains its regularity as long as it continues to become slower. On the sedative action being carried further, it rises in frequency, becomes irregular and intermittent, and still smaller and weaker." At this point, according to the author, it becomes exceedingly *irregular*, in frequency, force and volume—often fluctuating from one condition to another. Change of posture, too, sensibly affects it, precisely as when under the influence of digitalis—indeed, from Dr. Fleming's experiments, there is a striking analogy between the effects of Aconite and Digitalis on the pulse in all respects. He explains the influence of the Aconite on the heart by direct application to its inner surface, when conveyed to it with the blood; or, by its paralyzing influence when conveyed into its tissue by means of the coronary arteries.

He also maintains that the sedative action of the remedy on the vascular system is quite independent of its action on the nervous system: a position in which, it is probable, he will find many to dissent from him.

IV. ON THE RESPIRATORY SYSTEM. In every case in which the author inquired into the frequency of the respirations, he found them diminished, and that in proportion to the amount of the drug administered.

V. ON THE ALIMENTARY CANAL.—The first sensation usually experienced after taking a dose of Aconite, was an agreeable warmth in the mouth and stomach. In some instances the first few doses excited nausea and even vomiting, which soon ceased, in consequence of the stomach becoming accustomed to the use of the medicine. No action whatever seemed to be caused on the bowels; and, according to the author's experience, it may be continued many weeks (in medicinal doses) without impairment of the appetite.

VI. On the Secerning System.—Aconite seemed to exert but a very moderate influence ; and whatever effect was observed in any of the organs to follow its employment, seemed attributable entirely to its sedative effect on the vascular and nervous systems.

In reference to its *cumulative action*, Dr. F. declares that he has not met with any convincing evidence that Aconite is a cumulative remedy, although he confesses, that in two instances symptoms presented themselves which induced him to suspect that it is so. The readers of the *Medical Examiner* will recollect the mournful case of Dr. Male, contained in the October number of last year (1845) extracted from the Provincial Journal, in which death was induced, in all probability, by this very cause. Such was the opinion of Dr. Male himself, expressed before his decease, of the cause of his extreme illness, and of his medical attendant and the coroner's jury in regard to his death, and there can hardly be a doubt of its correctness. He commenced with five drops of the tincture two or three times a day, and gradually increased it to ten drops. The evidence of its cumulative action has likewise occurred to many practitioners who have tried it, so that there can be no doubt that great care should be observed in augmenting the dose, or of long persisting in its use.

From what has been stated, the opinion of the author of this treatise in regard to the *therapeutic action of the Aconitum Napellus* may readily be inferred. He looks upon it as powerfully and directly sedative in its action on the *nervous and vascular systems*, and therefore well adapted for the relief of neuralgic cases, and also in the management of inflammatory diseases, even to the extent of a substitute for blood-letting. "It relieves pain by diminishing the sensibility of the part, when applied topically, without acting on the brain ; or, when given internally, it may have the effect of rendering the brain more or less insensible to impressions conveyed to it by the sensory nerves."

As an *Anti-Neuralgic*, its action, in the opinion of Dr. F., is materially different from that of a mere anodyne, such as opium. It not only allays pain, but is capable of removing permanently the morbid condition of the nerve on which the pain depends. "I

have met, [he remarks,] with several cases of neuralgia, in which the individuals had, for weeks or months, been in the habit of procuring sleep, and a temporary cessasion of pain, by opiate draughts, and who, on using the Aconite, obtained permanent relief for the disease."

This, probably, is the experience of nearly every one who has experimented with a good article. As to its "*antiphlogistic*" action to the extent at any rate of superseding the lancet, the case is much more doubtful. We certainly have not a whit more confidence in it than the kindred article, digitalis; and we very much doubt whether the evidence of its efficacy in inflammatory diseases is at all greater than the latter.

The great uncertainty of procuring the article of a uniform strength, has limited its use in the treatment of disease far more than is desirable, especially in neuralgic cases. Now that the attention of the profession has been so emphatically called to it, it is to be hoped that greater pains will be taken by our apothecaries to be always supplied with the genuine medicine, prepared in a uniform way.

*Administration.*—The following are the author's remarks on the administration of the remedy, which, as they explain the manner and extent of his employment of it, are important to a right understanding of his practice.

"Exhaustion by alcohol is the best mode of obtaining an active preparation of the plant. Cold water takes up but little of its virtues; and the expressed juice contains only a portion of its active properties; while the latter are entirely destroyed by exposure of the drug, in any of its forms, to a high temperature. These circumstances are explained by the fact, that Aconitina, the active principle of the plant, is soluble in alcohol, but not in water, and is decomposed by much heat. *Boiling in water, if long continued*, is sufficient to deprive the plant of the power of producing numbness and tingling (Geiger); but Dr. Christison has ascertained that this property is not diminished by a heat of 212°, in drying the plant, or in preparing an extract from it.

"These facts fully account for the variable strength and frequent inertness of many of the preparations formerly in use, such as watery extracts, expressed and inspissated juices, &c.

"*Tinctura Aconiti.*—Take of root of A. Napellus, carefully dried and finely powdered, sixteen ounces Troy; rectified spirit, sixteen fluid ounces; macerate for four days; then pack into percolator; add rectified spirit until twenty-four ounces of tincture are obtained.

"It is beautifully transparent, of the colour of sherry wine, and the taste is slightly bitter.

"*Extractum Alcoholicum Aconiti.*—This is prepared by distilling, at a low temperature, the spirit from the tincture, until the consistence of an extract has been obtained. The process should be completed in a vapour bath.

"Its colour is dark brown, or almost black; it has an agreeable smell, and bitter taste. The dose is one-third of a grain, thrice daily, commencing with one sixth of a grain.

"I prefer the tincture for internal administration, from its greater uniformity of action. The method of administering it varies according to the object in view.

"As an *anodyne*, *anti-neuralgic*, and *calmative*, five minims ought to be given at first, three times daily, to be increased daily to the extent of one minim each dose, until the physiological effects described under the second degree of operation have been produced.

"As an *Antiphlogistic*, five minims ought to be given at first, and repeated in four hours; by which means the second degree of operation will, in all likelihood, have been induced. In order to sustain the sedative action thus developed, two and a half minims are to be given every three or four hours; or less frequently, according to the effect produced.

"Where this mode of administration is adopted, it is *absolutely necessary* that the patient should be seen, and his pulse examined, before the exhibition of each dose. When this cannot be done, the remedy may be given in the manner pointed out for its use as an anodyne and calmative.

"The *best method* of administering the remedy in *diseases of the heart*, is to give it in smaller doses than those recommended for its use as an anodyne, but more frequently repeated, as three or four minims five times daily.

"Sickness may be avoided or checked by an effervescing draught, administered with, or immediately after, the dose.

"The long-continued use of Aconite occasionally renders the patient less susceptible of its influence, in which case the dose must be gradually increased."

We again caution the reader against the direction contained in the last paragraph. Aconite is too powerful a medicine, and the evidence of its cumulative properties too well authenticated, to justify any one in running so great a risk.

The *tincture*, as prepared by Dr. Fleming, differs most essentially from that prescribed in the U. S. Pharmacopœia. The formula given in the latter is as follows: "Take of Aconite *four ounces;* Diluted Alcohol *two pints.* Macerate for fourteen days, express, and filter through paper"—or it may be made by the common process of *displacement*, using the same quantity of the Aconite for making two pints of the tincture. The dose of this is from twenty to thirty drops, three times a day, in water. It must also be observed that according to the U. S. process the dried *leaves* are employed in the preparation of the tincture, whereas Dr. Fleming uses the *root*, which he alleges is more powerful than the leaves.

---

*Handbuch der Heilmittellehre*, von Dr. Fr. Oesterlen, Professor der Medicin an der Universität Tübingen. Erste und zweite Lieferung. 8vo. pp. 1051. Tübingen, 1845.

*Manual of Materia Medica.* By Dr. Fr. Oesterlen, Professor of Medicine in the University of Tubingen. 8vo. pp. 1051. Tubingen, 1845.

*Nouveau Formulaire Magistral précédé d'une notice sur les Hôpitaux de Paris, de generalités sur l'Art de formuler, suivi d'un Précis sur les Eaux minérales naturelles et artificielles, d'un Mémorial Thérapeutique, de Notions sur l'Emploi des Contre-poisons et sur les Secours à donner aux Empoisonnés et aux Asphyxiés:* par M. Bouchardat, Docteur en Médecine et Agrégé de la Faculté de Médecine de Paris, Pharmacien en chef de l'Hotel-Dieu. 3eme. édit. enrichie de plusieurs médicaments nouveaux, tels que la digitaline, le valérianate de zinc, &c. 12mo. pp. 487. Paris, 1845.

*Much improved and extended—The Medical Formulary: being a Collection af Prescriptions derived from the writings and practice of many of the most eminent physicians in America and Europe; to which is added an appendix containing the usual dietetic preparations and antidotes for poisons: the whole accompanied with a few brief pharmaceutic and medical observations.* By Benjamin Ellis, M. D., Late Professor of Materia Medica and Pharmacy in the Philadelphia College of Pharmacy. Eighth edition, with numerous additions. By Samuel George Morton, M. D. 8vo. pp. 272. Philadelphia, 1846.

In our last number we alluded to the valuable results of the labours of German investigators whenever they are directed to the sciences of observation; and enumerated the nature of our indebtedness to them We do not think, that therapeutics is a branch that has been eminently benefited by their researches.

Something more than observation is required in it. So many disturbing influences are perpetually at work; so much sound reflection is needed in tracing effects accurately to their causes, that a simple observation of phenomena may lead to erroneous and often most irrational deductions. Judgment is eminently necessary to constitute the rational therapeutist, and this judgment is too often wanting; hence it is easy to record facts, but more difficult to see their relations, and to establish laws founded on such relations,—to form, in other words, a science from observed phenomena.

In every case of disease to which therapeutical skill has to be directed—some hypothesis—some ground of action based on observation and reflection must be established—some indication of treatment, in other words; otherwise there can be no reason why one article of the materia medica should be selected rather than another. The establishment of such hypothesis frequently demands not only accurate observation, but profound reflection and hence the cause of the deficiency of good therapeutists—whilst we have multitudes of so-called observers—in the ranks of the profession and out of it; for every Homœopathist and Thompsonian; every professed empiric, from Drs. Rock and Brodum downwards, has had no lack of facts—the results, it is believed by him, of positive experience—to fix the value of his procedures or nostrums.

The Manual of Materia Medica of Dr. Oesterlen is one of the best German works on the subject that have fallen under our notice. It is not based on the fashion of the day, which appears to consist in cramming into works on a specialty every thing of a collateral character that can be presumed to have any bearing upon it, and that may be found in treatises on other subjects. Dr. Oesterlen properly remarks, that the whole domain of the natural history of the materia medica is of inferior moment in a work of the kind. "Why," he asks, "should the practitioner or the student have to wade through preliminary observations on the chemical, botanical or pharmaceutical material, before he reaches the special object of his wishes—the action and therapeutical use of the remedial agent?" Neither the Germans nor the French have indulged this taste, and hence their works on the materiā medica are better adapted for the medical student. They are therapeutical rather than pharmaceutical.

Like most approved works on the materia medica, the one before us inquires first into the general properties of therapeutical agents; their *modus operandi;* the changes produced in the agents themselves by their operation; as well as in the organism and its parts, &c.; after which some observations are made on poisons and their mode of action; on the treatment of poisoning; the methods of administering medicinal agents, &c. &c.; on all which subjects the author appears to us to possess orthodox views, with occasional suggestions that belong somewhat to the realms of imagination rather than of exact observation. In regard to the uses of medicines according to age, the following table is given, which he properly regards as approximative only. Reckoning the dose for an adult to be 1, or 5 grains, the dose will be:—

| | | |
|---|---|---|
| Before the age of 1 year, | $\frac{1}{16}$ to $\frac{1}{8}$, | or $\frac{1}{4}$ to $\frac{1}{3}$ of a grain. |
| From 1 to 5 years, | $\frac{1}{6}$ to $\frac{1}{3}$, | or $\frac{1}{2}$ to $1\frac{1}{2}$ grain. |
| " 6 to 14, | $\frac{1}{2}$ to $\frac{3}{4}$, | or 2 to $3\frac{1}{2}$ grains. |
| " 15 to 20, | $\frac{4}{5}$ to 1, | or 4 to 5 grains. |

The following table may serve to regulate the dose according to the particular mode in which the agent has to be administered. If the dose of a medicine taken into the stomach be estimated at 1, or 5 grains,—as a general rule it may be when applied

| | |
|---|---|
| To the sound skin, | 5 to 8, or 25 to 30 grains. |
| To ulcers or suppurating surfaces, | 2 to 6, or 10 to 30 grains. |
| Endermically, | 2 to 3, or 10 to 15 grains. |
| In enemata, | 2 to 4, or 10 to 20 grains. |
| To the eye, | $\frac{1}{3}$ to 1, or 2 to 5 grains. |

p. 89.

Dr. Oesterlen correctly remarks, however, that all these rules must have numerous exceptions.

The classification which he adopts is the physiological—the best, we think, for a treatise on materia medica. We do not lay much stress on the particular classification that may be embraced by an author. It ought to be one, however, which will include most—if complete it ought to include all—of the articles of the materia medica. Dr. Oesterlen's main classes are: 1, Alteratives (*Alterantien.*) 2, Roborants or Tonics.

3, Excitants. 4, Acrids or Irritants. 5, Cerebro-spinants or Narcotics and Tetanics. 6, Purely toxical agents. 7, Nutrients and Dietetical agents; and 8, Physical agents—Imponderables—as Cold, Heat, Electricity and Magnetism. Well founded objections might be urged against this general arrangement—but we have not time; and if we had, the utilitarian would perhaps consider them to be scarcely worth the labour of recording.

The mode in which the author treats of the different articles, after describing the therapeutical action of the class, is as follows—taking iodine as an example. He gives: 1, Its physiological operation. 2, Lesions after death. 3, Treatment of poisoning by it. 4, Its therapeutical application when given internally. 5, Its external use. 6, Contraindications, and general rules for its use. He then passes to the particular preparations, on which he is somewhat brief. We take the following as a sample:—

"*Tinctura Iodii, Iodtinctur.*

"A solution of 48 grains of Iodine in an ounce of alcohol. Ph. Bor. 16 to 18 drops contain a grain of Iodine. This solution is often used internally, although it is not an entirely certain preparation. It very soon, for instance, undergoes a partial decomposition, forming hydriodic acid with traces of hydriodic ether, especially under the influence of light, by which it becomes weaker. It is more proper, therefore, that the tincture should always be freshly prepared, and not in large quantity.

"Dose. Five to ten drops twice a day, gradually increasing the quantity, in sugared water,—pure water precipitates the iodine in considerable quantity. Externally, like all the preparations of iodine, the tincture is used; especially to produce a more intense degree of action—as in lupus, sycosis, alopecia, tinea, and glandular tumefactions; in diffuse inflammation of the subcutaneous cellular tissue, with tendency to gangrene (pseudo-erysipelas) and whitlow. Its action can be diminished by the addition of alcohol. It is seldom employed in baths." p. 327.

The above extract will show, that the author is too brief; yet his work is unquestionably one of the most satisfactory we have seen from Germany, and we can therefore recommend it to the reader of German.

M. Bouchardat's researches and publications in the domain of pharmacology have gained him no little reputation. In a former number of this journal we drew attention to one of his annual labours—the *Annuaire de Thérapeutique*—which still holds its useful course;—the part for 1846, with a supplement, having already made its appearance. The *lengthy* title at the head of this article indicates the diversified contents of the "Magistral Formulary," the utility of which is sufficiently attested by the fact, that it is now in its third edition, and brought up to the existing condition of medicine and pharmacy. We have more than once said, that we doubt the ultimate value to practitioners of these formularies; or rather, we question whether there be not many important countervailing objections that may be brought against them. They certainly are apt to teach the young practitioner, that a precise and unvarying combination of agents is demanded for disease, which is itself ever varying; and induce him to have confidence in set and special formulæ as adapted for the removal of special morbid conditions. They are books that ought to be consulted occasionally,—assuredly not to be made the subjects of daily reference. M. Bouchardat affirms, that a magistral formulary is an indispensable accompaniment to a treatise on materia medica and pharmacy. (p. 11.) As examples of the mode of compounding and combining medicines, a collection of judicious formulæ, like the one, for example, appended to M. Oesterlen's work, is certainly advisable; but we much doubt, whether the same good can be ascribed to such works as those of M. Bouchardat and Dr. Ellis, in which are collected together a multitude of formulæ, to many of which the term "judicious" might be appropriately applied, whilst to others the epithet would certainly be most inappropriate. Many would seem, indeed, to have obtained admission, less from their intrinsic value, than as personal compliments from the author of the formulary to the particular prescriber.

From the note on the Hospitals of Paris prefixed to M. Bouchardat's work we glean the following facts, which may be interesting to most of our readers. The Hotel Dieu has 750 beds; and a provisional hospital, called *Annexe de l'Hôtel Dieu*, 400. The physicians are MM. Récamier, Husson, Guéneau de Mussy, Caillard, Honoré, Magendie, Jadioux, Louis, Chomel, Rostan, Legroux, Requin and Sandras; the surgeons, MM. Roux, Breschet and Blandin; the *pharmaciens*, MM. Bouchardat and Lortier. The Hôpital de la Pitié has 600 beds; the physicians are MM. Serres, Clement, Piorry, Gendrin and Bouvier; the surgeons, Lisfranc and Bérard; and the *pharmacien*, M. Guiart. The Hôpital de la Charité has 492 beds; the physicians are MM. Fouquier, Rayer, Andral, Bouillaud and Cruveilhier; the surgeons, MM. Velpeau and Gerdy; and the *pharmacien*, M. Quevenne. The Hôpital Saint Antoine has 300 beds: the physicians are MM. Kapeler, Guérard and Piedagnel; the surgeons, MM. Bérard ainê and Malgaigne; the *pharmacien*, M. Fordos. The Hôpital Necker can contain 400 patients: the physicians are MM. Bricheteau, De la Roque and Trousseau; the surgeon is M. Lenoir; the lithontriptist, M. Civiale. The Hôpital Cochin has 100 beds: the physicians are MM. Blache and Briquet; the surgeon, M. Michon. The Hôpital Beaujon will contain 500 beds: the physicians are MM. Renauldin and Martin-Solon; the surgeons are MM. Marjolin, Laugier and Robert; *pharmacien*, M. Chatin.

These are the general hospitals; but there are other special hospitals in that great metropolis;—in other words, establishments intended for a particular class of diseases, or for such as have to be subjected to a particular regimen. The Hôpital Saint Louis has at present 800 patients: its physicians are MM. Lugol, Emery, Cazenave, Devergie and Gibert; the surgeons, MM. Jobert and Boyer; the *pharmacien*, M. Foy. The Hôpital des Cliniques has 135 beds: the surgeons are MM. Cloquet and Paul Dubois,—the latter for the obstetrical cases. The Hôpital des Vénériens ou du Midi has 400 beds: the physician is M. Puche; the surgeons are MM. Ricord and Vidal; the *pharmacien* is M. Grassi. The Hôpital de Lourcine received, in 1840, 2,083 female syphilitic patients: the physician is M. Bazin; the surgeons are MM. Denonvilliers and Huguier; the *pharmacien* is M. Lutz. The Maison Royal de Santé, formerly Hospice Dubois, has received, in the year, 1,509 patients; the physicians are MM. Dumeril, Hervez and De Chegoin; the surgeon is M. Monod; the *pharmacien*, M. Ducom. The Hôpital des Enfans Malades has 560 beds: the physicians are MM. Jadelot, Guersent, Bonneau and Baudelocque; physician orthopedist, M. Guérin; surgeon, M. Guersant fils; the *pharmacien*, MM. Bataille. The Hospice de la Maternité ou d'Accouchement has 433 beds: the physicians are MM. Moreau and Gérardin; the surgeons are MM. Paul Dubois and Danyau; the *pharmacien* is M. Hainque. The

Hospice des Enfans Trouves ou d'Allaitement admitted, in 1837, 5,467 cases: the physician is M. Baron; physician orthopedist, M. Bouvier; the surgeons are MM. Thévénot de Saint Blaise, and A. Auvity. The Infirmary of the Hospice de la Vieillesse (Femmes) Saltpêtrière has 400 beds: the physicians are MM. Prus and M. N. Nonat; the surgeon is M. Manec; the *pharmacien*, M. Fermond. The number of insane, idiotic, and epileptic in this immense establishment is from 1000 to 1200; and the physicians to this department are MM. Falret, Mitivié, Lelut, Trélat and Baillarger. The Hospice de Bicêtre receives aged males, and insane: the physicians to the aged sick are MM. Rochoux and Guillot-Natalis; the surgeon is M. Nélaton: the physicians to the insane are MM. Leuret, Voisin, Moreau and Delasieuve.

These are the chief Parisian charitable establishments, connected directly or indirectly with the medical profession. It would, however, be even more idle for any one to suppose, that, by visiting that metropolis for a year or two, he could reap the advantages of clinical instruction from all of them, than for him to presume that, from seeing a list of the hospitals and dispensaries in operation in New York, he could avail himself of all or even of several of them. There are but few which are much followed by the student even in Paris, where the hours for hospital attendance are placed so early that they do not interfere with the regular lectures at the Ecole de Médecine. Where the time of the student is largely occupied, as it is in our colleges during the winter course of lectures, and the hour for clinical attendance at the hospitals and dispensaries is established at a later hour—and it seems to be impracticable, consistently with our habits, to fix it earlier in the day—much time cannot possibly be given to clinical attendance; hence it generally happens, that the student attaches himself to one hospital only, and if he employs his time profitably it is enough, provided—as in Philadelphia—he pays adequate attention to the valuable—we might almost say invaluable—clinics, which are held thrice a week at the two larger schools:—that at the Jefferson Medical College at such an hour as not to interfere with clinical attendance at the Pennsylvania Hospital.

We could not—if we desired to do so—specify individual articles in M. Bouchardat's Formulary. It is naturally—

(we might say from the general custom with French authors)—confined mainly to the formulæ of his own countrymen. In the pharmaceutical portion it is full; and in the therapeutical concise. It is—as before said—up to the day, and therefore must be a useful addition to the library of the physician and pharmaceutist.

Ellis's Formulary has been long before the profession. It is now in its eighth edition, and has, consequently, met with much favour. We are told by the editor, Dr. Morton, that to the present edition many new and important additions have been made, "derived from recent discoveries in chemistry, and from the observation and experience of professional men in various parts of the world. Every section of the work has been scrupulously examined and extended, and the nomenclature revised in accordance with that of the United States *Dispensatory*." Dr. Morton doubtless means *Pharmacopœia*,—as the latter is the national work; the former a private production. Certainly, the nomenclature of the former editions was most heterogeneous. The present is greatly improved, but still it might be *emendata et expurgata*. We would suggest, for example, that in accordance with custom the drug should have precedence in such cases as Pulveris Valerianæ, Pulveris Acaciæ, which we take at random. Many typographical errors also still exist. In the same page from which one of the above examples is taken, we have *mucilaginous* for *mucilaginis*, (p. 172;) and turning at random to the very last page of the work, (260) we see *blaterium* for *elaterium:* these, too, meet us without seeking for inaccuracies; and, on the contrary, with an ardent desire to praise rather than to censure. Still, we can feel with the Editor that he has spared no pains "to impart to this work the comprehensiveness and accuracy which are due to the nature of the undertaking and the importance of the subject."

*The Seven Books of Paulus Ægineta. Translated from the Greek, with a Commentary embracing a complete view of the knowledge possessed by the Greeks, Romans and Arabians, on all subjects connected with Medicine and Surgery.* By FRANCIS ADAMS. Vol. 2; pp. 511. London, 1846.

*The Writings of Hippocrates and Galen, epitomized from the Original Latin Translations.* By JOHN REDMAN COXE, M. D., Member of the Batavian Society of Sciences at Haarlem; of the Royal Medical Society, and of the Royal Society of Sciences of Copenhagen, &c. 8vo. pp. 681. Philadelphia, 1846.

These works have reached us so recently, that we have not opportunity to give them more than a passing notice. They belong to the class of productions that may be ranked amongst luxuries; and yet luxuries, which every one ought to strive to possess.

The first volume of the works of Paulus of Ægina we heralded on its appearance some time ago. It is from the press of the Sydenham Society of London, and forms, we presume, the last volume for the year;—the three volumes now issued being:—the one before us—the second volume of Simon's Animal Chemistry, and Hasse's Pathological Anatomy. In the pages of some of the hebdomadals of Great Britain we have seen the Society ridiculed for publishing works of so little present "practical" value as those of Paulus of Ægina; and yet it appears to us that none could come more fairly within the scope of that most useful institution. Paulus affords in the text his own views on the different departments of medical science; and the erudite commentaries of Mr. Adams furnish not only the opinions of contemporaries and of those of his predecessors, but also references for the inquirer to the precise works, and portions of those works, should he desire to obtain further information. The volume before us comprises the fourth, fifth and sixth books of Paulus. One more will complete the work; and we are free to say, that we have not added to our own library any publication for years with more satisfaction than this. It is a most admirable book of reference for the "knowledge possessed by the Greeks, Romans and Arabians on all subjects connected with medicine and surgery,"

and we cheerfully award to the Sydenham Society our thanks for having made it one of their selections.

The work of Dr. Coxe was announced by us some time ago as in preparation. It is delightful to see one who has passed through so many summers, and has long been enjoying the otium that ought to follow a life well spent, still retaining his fondness for studies which engaged his mind half a century before, with sufficient mental and corporeal energy to undertake and execute in so creditable a manner a work like the present.

Dr. Coxe is essentially *laudator temporis acti;* and the motto which he has selected, "multa renascentur," shows his impression in regard to reputed novelties. Over and over again, indeed, in different portions of the work, he considers, that most of the discoveries—as they have been regarded by the moderns—were known in antiquity; or that germs existed then, which have not required much art to develope. It is obvious that full justice could not possibly be given to the voluminous works of these illustrious ancients in a publication like the present. "The only object of the Editor," says Dr. Coxe, "is that of affording a slight view of the subject-matter of the extensive treatises of these venerable writers; too slight, indeed, to constitute even an imperfect idea of a tithe of their merits, yet enough, he hopes, to give an impulse to a further research of their interesting pages." "The gratification," he adds, "I have experienced in looking over the writings of these pioneers of medicine has led me to believe, that even this imperfect exposition may be acceptable to many, and that more especially since few are likely to possess them in their complete and perfect form; yet it is necessary again to repeat, that to estimate the whole by this defective abstract, would be like one who judged of the character of a building by examining a brick which formed a fractional part of it. I have therefore to request all due allowance for this attempt to introduce to my contemporaries a few faint traces of their medical progenitors, who lived two thousand years before them." p. v.

We would not cavil unnecessarily at the enthusiasm with which the veteran author regards the illustrious ancients—especially Galen, to whom he refers discoveries and germs of discoveries that may well be contested. The germ of the discovery of the steam engine might be placed in the observance of the explosive force of steam pent up in a vessel; yet how different is this from the application of that same power to propulsion; and if germs of discoveries are to have the credit of discoveries themselves, it will not be easy for us to meet with one which may fairly date in modern periods. It has been a favorite topic with Dr. Coxe to investigate the claims of Harvey to the discovery of the circulation of the blood, and the result of his investigations has led him to deny priority to that distinguished individual. In his commentaries on the 18th book of Galen—*De Pulsuum Usu*—the intent of which book seems to be, he says, "to show that the use of the pulse is that of preserving the innate heat, and of conveying the animal spirits to every part," [!] Dr. Coxe refers to the fact, that Galen was aware of the communication between the arteries and the veins, and exclaims:—"Yet Harvey is regarded as the full discoverer of the circulation, and all his predecessors are alike consigned to oblivion, nay, in many cases, to contempt and obloquy! A *complete* translation of the works of Galen would effectually prove the frauds that have been perpetrated to support the honour of the British nation, which would be tarnished by the abstraction of those laurels that have been so unjustly awarded to a man considered as the glory of their country!" p. 537. Yet French and German writers—nay the writers of all countries—would scarcely unite to glorify the British nation through their illustrious countryman, if he were not entitled to some unusual credit in this matter. Nor is it exact history to say, that all the merit of the discovery of the circulation has been awarded to Harvey. Great merit has been assigned to Galen by all writers. All, too, ascribe due credit to Servetus, Cæsalpinus, Realdus Columbus and others for their correct appreciation of the pulmonic or lesser circulation; and it has been imagined, that they possessed some notion of the greater. Whether this were the fact or not, all nations unite in awarding to Harvey the merit, if not of entire originality—and to this he never laid claim—of at least having fairly and well described the whole circulatory movement. We confess then our entire belief, that the honour of the discovery is essentially his, and upon the same principle, that we award the discovery of the application of steam to the propulsion of vessels to Fulton. The obloquy which the promulgation of Harvey's views drew upon him is strong confirmation of this, as well as the bitter opposition that was made to their reception. "Nothing," says a distinguished writer—Purkinje—and a German, be it observed—"proves more significantly that until Harvey the circulation was not discovered, than the efforts of his opponents to decry it, or to contest his priority. His opponents were Primerose, Parisanus, Casp. Hofmann, Vesling, Gassendi, Plempius, Riolan and others. Yet before his death (1657) Harvey saw the complete establishment of his doctrine, and his opponents silenced one after another."*

We do not know, that this last assertion tallies exactly with the remark of Hume,—that no physician in Europe, who had reached forty years of age, ever to the end of his existence adopted Harvey's notion of the circulation; and Harvey's practice in London diminished extremely for a time from the reproach drawn upon him for that great and signal discovery! Surely this reproach from his *own countrymen* would not have visited him, had he not been regarded as a real discoverer, and not the mere promulgator of views which had been long, as long even as the time of Galen, entertained by others!

But we have already said more than we designed. Although we may differ from the veteran author as to the relative merits of the ancients and moderns, we agree with him, that many who pride themselves at the present day in being *original,* could readily find in the works of the ancient writers that they had been anticipated ages ago: and we cordially recommend this book to all who take a delight—and we hope there is no one who does not—in the classic productions of the great Fathers of our art.†

*Art. Circulatio Sanguinis in Encyclopäd. Wörterb. der Medicinisch. Wissensch. vii. 699, Berlin, 1831.

† We may ask, by the way, on what sufficient authority, the author writes Fœsius for Foësius?

*Small Books on Great Subjects.* No. 2. *On the Connection between Physiology and Intellectual Science,* (from the second London Edition.) 12mo. pp. 85. Philadelphia, 1846. No. 3. *On Man's Power over himself to control Insanity.* 12mo. pp. 54. Philadelphia, 1846. No. 4. *An Introduction to Practical Organic Chemistry with References to the Works of Davy, Brandes, Liebig, &c.* 12mo. pp. 66. Philadelphia, 1846.

These small books are samples of a series which has appeared in England, met with much success, and been re-printed in this country. Being brief, they can be perused and studied even in the dog-days; and they have accordingly been read by great numbers, who might have been deterred had their magnitude been greater. "The Editors of the 'Small Books' have already had occasion to notice the almost unexpected success which has attended their undertaking: of course this excites a corresponding zeal to deserve it, and they flatter themselves, that the increased quantity of letter press in the later numbers of the series will be such as to meet the wishes of those who before considered the price too high for the size of the books." We extract this from the preface to the second edition of the first of the "small books" whose titles are given above. It, as well as the second, is from the pen of the Rev. John Barlow, Secretary of the Royal Institution of Great Britain; and both contain the substance of a communication made to the members of the Royal Institution at one of their Friday evening meetings. Both, too, we are told, are offered to the public at the desire of many who were present on those occasions; and the publication of one of them was required by the President of the Institution.

The object of the Introduction to Practical Organic Chemistry—as stated by its author—is "to give a few of the great principles, which a very interesting portion of modern science is grounded upon; and by omitting technical details, to make it available to the scientific reader."

We can recommend all these Essays. They are well adapted to the purpose had in view in their publication; and we may say as much for the others of the series that have already been published by Messrs. Lea & Blanchard. They are, respectively,

No. 1. Philosophical Theories and Philosophical Experience, (from the second London Edition), No. 5. A brief view of Greek Philosophy up to the age of Pericles; and No. 10, The Principles of Criminal Law.

---

*French Cookery. The Modern Cook, a Practical Guide to the Culinary art in all its Branches, adapted as well for the largest establishments as for the use of private Families.* By Charles Elmé Francatelli, Pupil of the celebrated Carême, and late Maître d'Hôtel and Chief Cook to Her Majesty the Queen: with numerous Illustrations. Large 8vo. pp. 540. Philadelphia, 1840.

In a former number of this journal, when noticing Miss Acton's "Modern Cookery," we alluded to the dignity of the *Ars Coquinaria* in ancient as well as in modern times; to its value also to the physician, and to the large part which he had taken in extending its domains. The work before us is one of the most elaborate that has issued from the press, and shows that its author is fully master of the art, if the fact that "he has had the good fortune to be *chef-de-cuisine* to the Earl of Chesterfield, Lord Kinnaird, and to Sir W. Massey Stanley, Bart., and Rowland Errington, Esq., at Melton Mowbray," and that he had served "as chief cook, and maître d'hôtel to her most gracious Majesty the Queen," did not amply establish it.

It appears to us, that the work ought to form part of every domestic establishment; for granting that many, if not most, of the dishes may be too *recherchés* and expensive for ordinary use, the directions furnish ideas to the good housewife which cannot fail to be valuable. Moreover, Mr. Francatelli has given several preparations which are interesting to the physician;—all are of course so to the hygienist. For example, there is a heading for *Medicinal Broths and Consommés for Invalids;* comprising Plain Chicken broth; Pectoral do. do.; Mutton do.; Beef tea; Cray fish broth; Decoction of Snails; and Mucilaginous chicken broth. His recipe for beef tea is as follows:

"Take two pounds of the lean part of the gravy-piece of beef, and carefully pare away every portion of fat, skin, or sinew; cut this into small and square pieces the size of a nut; put the beef into a stewpan capable of containing two quarts, and pour three pints of boiling water upon it; add a little salt, put it on the stove fire, and as soon as it boils skim it, and then remove it to the side of the stove, to continue boiling gently for an hour, after which the Beef Tea should be strained through a napkin for use." p. 64.

The following article is not much known in this hemisphere, but is common in some parts of the Old World.

" *Decoction of Snails for Inveterate Coughs.*

Take two dozen garden snails, add to these the hind quarters only of two dozen stream frogs, previously skinned; bruise them together in a mortar, after which put them into a stewpan with a couple of turnips chopped small, a little salt, a quarter of an ounce of hay saffron, and three pints of spring water. Stir these on the fire until the broth begins to boil, then skim it well, and set it by the side of the fire to simmer for half an hour; after which it should be strained by pressure through a table cloth into a basin for use. The broth, from its soothing qualities, often counteracts successfully the straining effects of a severe cough, and alleviates more than any other culinary preparation the sufferings of the consumptive." p. 65.

There is a section also on " *Panadas and light soups for Infants and Invalids*"—comprising Chicken Panada, Pheasant or Partridge Panada, chicken or game Custards, Venison Panada, Nutritious Liquid Custards of Chickens, do. do. of Game and Ceylon moss; Gelatinous Chicken Broth. The first of these might readily tempt the appetite of a valetudinarian.

" *Chicken Panada.*

Roast off a young fowl, take all the white parts and pound them with the crumb of a French roll soaked in broth; dilute these with a little chicken broth (made from the remains of the roasted fowl) to the consistency of a soft batter or creamy substance; pass it through a tammy as in preparing any other punêe. Previous to stewing this *panada* it should be moderately warmed and put into custard cups. In the composition of every sort of dietetic preparation for the use of infants and invalids, it is strictly necessary to avoid the use of herbs, vegetables and spices; even salt should be used sparingly." p. 119.

We should differ, however, in regard to the last article, which is a sort of natural condiment, and always agreeable to the child.

One more formula we may add.

" *Ceylon Moss Gelatinous Chicken Broth.*

Cut a fowl into four parts, take out the lungs, and wash it thoroughly, place it in a stewpan with four ounces of prepared Ceylon Moss, adding three pints of water and a little salt; having boiled the broth for three quarters of an hour by the side of a stove fire, pass it through a napkin, and serve it in a caudle cup to the invalid." p. 120.

These extracts will be sufficient to show the useful nature of the work.

---

*Human Physiology. With three hundred and sixty-eight illustrations.* By ROBLEY DUNGLISON, M. D., Professor of the Institutes of Medicine in Jefferson Medical College of Philadelphia; Secretary to the American Philosophical Society, etc., etc. *Sixth Edition. Greatly Improved. In two volumes* 8vo. Lea & Blanchard: Philadelphia, 1846.

Those who have been accustomed to consult the former editions of this work, know with how much care and accuracy every fact and opinion of weight, on the various subjects embraced in a treatise on physiology, is collected and arranged, so as to present the latest and best account of the science. To such we need hardly say, that, in this respect, the present edition is not less distinguished than those which have preceded it. In the two years and a half which have elapsed since the last or fifth edition appeared, nothing of consequence that has been recorded seems to have been omitted. Upwards of seventy illustrations have been added, and many of the former cuts have been replaced by others of better execution. These mostly represent the minute structures as seen through the microscope, and are necessary for a proper comprehension of the modern discoveries in this department.

In conclusion, we cannot better express our estimation of the value of this edition of our learned author's work, than by citing the language employed by an able critic in a foreign journal, when speaking of a former edition: "To those who desire a work on physiology more extended than that of Dr. Carpenter, and more readable than that of Professor Müller, we can strongly recommend this treatise of Dr. Dunglison's. The large mass of references which it contains, renders it a most valuable bibliographical record, and bears the highest testimony to the zeal and industry of the author."*

* British and Foreign Medical Review, October, 1844.

